# Supplementary material for: The Evolution and Characterization of the RNA Interference Pathways in Lophotrochozoa
Source: Genome Biol Evol. 2024 May 7;16(5):evae098. doi: 10.1093/gbe/evae098 (PMC11114477; doi:10.1093/gbe/evae098)
Supplement: evae098_Supplementary_Data [file evae098_supplementary_data.zip › Supplementary_materials.zip!/selected_miRNAs/NoGe_selected_miRNAs/mirdeep_pdf_files/NMRB01000087.1_Notospermus_geniculatus_strain_Ushimado_scaffold87,_whole_genome_shotgun_sequence_3152.pdf]

Provisional ID : NMRB01000087.1_Notospermus_geniculatus_strain_Ushimado_scaffold87,_whole_genome_shotgun_sequence_3152
Score total : 2852.2
Score for star read(s) : 3.9
Score for read counts : 2843.4
Score for mfe : 1.8
Score for randfold : 0
Score for cons. seed : 3
Total read count : 5589
Mature read count : 5583
Loop read count : 0
Star read count : 6

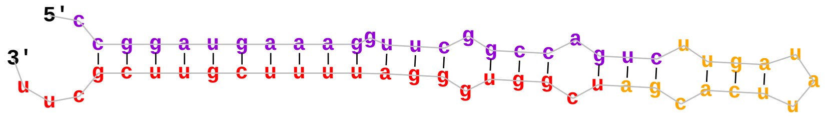

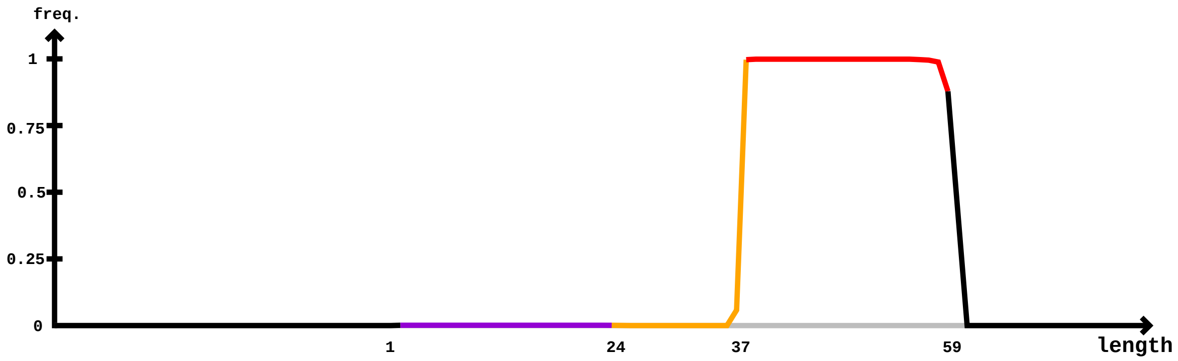

Star Mature

```
5'- cucaaacaaggauggugagcgcuacuuguuucguccggaugaaagguucggccagucuugauauucacgaucggugggauuuucguucgcuuggaauaaguagcaguccuuc  -3'  obs
    cucaaacaaggauggugagcgcuacuuguuucguccggaugaaagguucggccagucuugauauucacgaucggugggauuuucguucgcuuggaauaaguagcaguccuuc       exp
    .......((((((.......(((((((((((((..((((((((((.(((.(((.(((.(((...))).))).))).)))))))))))))..))))))))))))).)))))).  reads  mm  sample
    ......................................................................ucggugggauuuucguucgc......................  5      0   Mm1
    ......................................................................ucggugggauuuucguuAgcuu....................  1      1   Mm1
    ......................................................................ucggugggauuuucguucgcuu....................  100    0   Mm1
    ......................................................................ucggugggauuuucguuGgcuu....................  1      1   Mm1
    ......................................................................ucggugggGuuuucguucgcuu....................  1      1   Mm1
    ......................................................................ucggugggauuuucguucgcuuU...................  16     1   Mm1

    ..................................ccggaugaaagguucggccagu........................................................  1      0   Mm2
    ..................................ccUgaugaaagguucggccaguc.......................................................  1      1   Mm2
    .....................................................................aucggugggauuuucguucgc......................  1      0   Mm2
    .....................................................................aucggugggauuuucguAcgcu.....................  1      1   Mm2
    .....................................................................aucggugggauuuucguucgcu.....................  20     0   Mm2
    .....................................................................aucggugggauuuucguucgcG.....................  1      1   Mm2
    .....................................................................aucggugggauuuucguucgcuu....................  1      0   Mm2
    .....................................................................aucggugggauuuucguucgcuuU...................  1      1   Mm2
    ......................................................................ucggugggauuuucguuc........................  1      0   Mm2
    ......................................................................ucggugggauuuucguucgc......................  2      0   Mm2
    ......................................................................ucggugggauuuucguucgcu.....................  63     0   Mm2
    ......................................................................ucggugggauuuucguGcgcu.....................  1      1   Mm2
    ......................................................................ucggugggauuuucguucgcG.....................  2      1   Mm2
    ......................................................................ucggugggauuuucguuUgcu.....................  1      1   Mm2
    ......................................................................ucggugggauAuucguucgcu.....................  1      1   Mm2
    ......................................................................ucggugggauuuucgCucgcu.....................  1      1   Mm2
    ......................................................................ucggugggauuuucguGcgcuu....................  3      1   Mm2
    ......................................................................ucggugggauuuuUguucgcuu....................  1      1   Mm2
    ......................................................................ucggugggauuuucguucgcuG....................  7      1   Mm2
    ......................................................................ucggugggaAuuucguucgcuu....................  1      1   Mm2
    ......................................................................ucggugggauuuucguAcgcuu....................  2      1   Mm2
    ......................................................................ucggugggauuuucguCcgcuu....................  5      1   Mm2
    ......................................................................ucggugggauGuucguucgcuu....................  1      1   Mm2
    ......................................................................ucggugAgauuuucguucgcuu....................  1      1   Mm2
    ......................................................................ucggugggauuuucguuUgcuu....................  8      1   Mm2
    ......................................................................ucUgugggauuuucguucgcuu....................  1      1   Mm2
```

```
                                  Star                                Mature
cucaaacaaggauggugagcgcuacuuguuucguccggaugaaagguucggccagucuugauauucacgaucggugggauuuucguucgcuuggaauaaguagcaguccuuc
......................................................................ucggugggGuuuucguucgcuu....................    1     1    Mm2
......................................................................ucggugggauuuucguucgcuu....................    814   0    Mm2
......................................................................ucggugggauCuucguucgcuu....................    1     1    Mm2
......................................................................Ncggugggauuuucguucgcuu....................    1     1    Mm2
......................................................................ucggugggauuuucguuAgcuu....................    6     1    Mm2
......................................................................ucggugggauuuucguucgcuA....................    1     1    Mm2
......................................................................ucggCgggauuuucguucgcuu....................    1     1    Mm2
......................................................................ucgguAggauuuucguucgcuu....................    1     1    Mm2
......................................................................ucggugggauuuucguuGgcuu....................    4     1    Mm2
......................................................................ucggugggauuuucUuucgcuu....................    1     1    Mm2
......................................................................ucggugggauuuucguucgcuug...................    1     0    Mm2
......................................................................ucggugggauuuucguucgcuuA...................    8     1    Mm2
......................................................................ucggugggauuuucguucgcuuU...................    259   1    Mm2
......................................................................ucggugggauuuucguucgcuuC...................    2     1    Mm2
.......................................................................cggugggauuuucguucgcuu....................    1     0    Mm2
.......................................................................cggugggauuuucguucgcuuU...................    1     1    Mm2

.....................................................................aucggugggauuuucguCcgcu.....................    1     1    Fm2
.....................................................................aucggugggauuuucguucgcG.....................    1     1    Fm2
.....................................................................aucggugggaGuuucguucgcu.....................    1     1    Fm2
.....................................................................aucggugggauuuucguucgcu.....................    31    0    Fm2
.....................................................................aucggugggauuuucguucgcuu....................    25    0    Fm2
......................................................................ucggugggauuuucguucgcG.....................    1     1    Fm2
......................................................................ucggugggauuuucguCcgcu.....................    1     1    Fm2
......................................................................ucggugggauuuucguucgcu.....................    16    0    Fm2
......................................................................ucggugggauuuuAguucgcuu....................    1     1    Fm2
......................................................................ucggugggauuuucguCcgcuu....................    2     1    Fm2
......................................................................ucggugggauuuucguucgcuu....................    459   0    Fm2
......................................................................ucggugggauuuucguucgcuA....................    10    1    Fm2
......................................................................ucggugggauuuucguuUgcuu....................    3     1    Fm2
......................................................................ucggugggauGuucguucgcuu....................    1     1    Fm2
......................................................................Ncggugggauuuucguucgcuu....................    1     1    Fm2
......................................................................ucggugggGuuuucguucgcuu....................    1     1    Fm2
......................................................................ucggugggauuuucguuGgcuu....................    1     1    Fm2
......................................................................ucggugggauuuucguuAgcuu....................    1     1    Fm2
......................................................................ucgguUggauuuucguucgcuu....................    2     1    Fm2
......................................................................ucggugggauuuucguucgcuG....................    2     1    Fm2
......................................................................ucggugggauuuucguGcgcuu....................    2     1    Fm2
......................................................................ucgguAggauuuucguucgcuu....................    1     1    Fm2
......................................................................ucggugggauuuucguucgcuuU...................    307   1    Fm2
......................................................................ucggugggauuuucguucgcuuA...................    31    1    Fm2
.......................................................................cggugggauuuucguucgcuuU...................    6     1    Fm2

..................................ccggaugaaagguucggccaguc.......................................................    4     0    Fm1
.....................................................................aucggugggauuuucguucgcu.....................    21    0    Fm1
.....................................................................aucggugggauuuucguucgcG.....................    1     1    Fm1
.....................................................................Cucggugggauuuucguucgcuu....................    1     1    Fm1
.....................................................................aucggugggauuuucguucgcuu....................    1     0    Fm1
.....................................................................aucggugggauuuucguucgcuuU...................    2     1    Fm1
......................................................................ucggugggauuuucguuA........................    1     1    Fm1
......................................................................ucggugggauuuucguuc........................    6     0    Fm1
......................................................................ucggugggauuuucguucgc......................    2     0    Fm1
......................................................................ucggugggauuuucguucgcu.....................    63    0    Fm1
......................................................................ucggugggauuuucguuUgcu.....................    1     1    Fm1
......................................................................ucggugggauuuucguuAgcu.....................    3     1    Fm1
......................................................................ucgguggAauuuucguucgcuu....................    1     1    Fm1
......................................................................ucgguggCauuuucguucgcuu....................    1     1    Fm1
......................................................................ucggugggauuuucguuGgcuu....................    3     1    Fm1
......................................................................ucggugggauuuuUguucgcuu....................    1     1    Fm1
......................................................................ucggugggaGuuucguucgcuu....................    1     1    Fm1
......................................................................Ncggugggauuuucguucgcuu....................    1     1    Fm1
......................................................................ucggugggauuuucguuUgcuu....................    2     1    Fm1
......................................................................ucggugggauuuucguCcgcuu....................    3     1    Fm1
......................................................................ucggugggauuuucguucgcuG....................    1     1    Fm1
......................................................................ucggugggauuuucguAcgcuu....................    1     1    Fm1
......................................................................ucggugggauAuucguucgcuu....................    3     1    Fm1
......................................................................ucggugggauuuucguuAgcuu....................    5     1    Fm1
......................................................................ucgguNggauuuucguucgcuu....................    1     1    Fm1
......................................................................ucggugggauuuucguGcgcuu....................    2     1    Fm1
......................................................................ucggugggauuuucguucgcuA....................    1     1    Fm1
```

Star **Mature**

```
cucaaacaaggauggugagcgcuacuuguuucguccggaugaaagguucggccagucuugauauucacgaucggugggauuuucguucgcuuggaauaaguagcaguccuuc
......................................................................ucggugggauGuucguucgcuu....................   1     1   Fm1
......................................................................ucggugggauuuucguucgcGu....................   2     1   Fm1
......................................................................ucggugggauuuucguucgcuu....................   680   0   Fm1
......................................................................ucggugggauuuuAguucgcuu....................   1     1   Fm1
......................................................................ucggugCgauuuucguucgcuu....................   1     1   Fm1
......................................................................ucUgugggauuuucguucgcuu....................   3     1   Fm1
......................................................................ucggugggauuuucguucgcuuU...................   195   1   Fm1

.....................................................................aucggugggauuuucguucgc......................   1     0   Mm3
.....................................................................aucggugggauuuucguucgcu.....................   30    0   Mm3
.....................................................................aucggugUgauuuucguucgcu.....................   1     1   Mm3
.....................................................................aucggugggauuuucguAcgcu.....................   1     1   Mm3
.....................................................................Cucggugggauuuucguucgcuu....................   1     1   Mm3
.....................................................................aucggugggauuuucguucgcuu....................   4     0   Mm3
.....................................................................aucggugggauuuucguucgcuuU...................   1     1   Mm3
......................................................................ucggugggauuuucguuc........................   1     0   Mm3
......................................................................ucggugggauuuucguucU.......................   1     1   Mm3
......................................................................ucggugggauuuucguucg.......................   2     0   Mm3
......................................................................ucggugggauuuucguucgc......................   7     0   Mm3
......................................................................ucUgugggauuuucguucgcu.....................   2     1   Mm3
......................................................................ucggugggauuuucguuAgcu.....................   1     1   Mm3
......................................................................ucggugggauuuucguucgcG.....................   1     1   Mm3
......................................................................ucggugggauuuucguucgcu.....................   57    0   Mm3
......................................................................ucggugggauuuucguuGgcu.....................   1     1   Mm3
......................................................................ucggugggauuuucguCcgcuu....................   2     1   Mm3
......................................................................ucggugggauuuucguucgcuA....................   1     1   Mm3
......................................................................ucggugggauuuucguuGgcuu....................   2     1   Mm3
......................................................................ucAgugggauuuucguucgcuu....................   1     1   Mm3
......................................................................ucggugggauuuucguGcgcuu....................   2     1   Mm3
......................................................................ucggugggauuuucguucgcuC....................   1     1   Mm3
......................................................................ucggugggauuCucguucgcuu....................   1     1   Mm3
......................................................................ucggugggauuuucguAcgcuu....................   2     1   Mm3
......................................................................ucggugggauuuucguucgcuu....................   692   0   Mm3
......................................................................ucggugggauGuucguucgcuu....................   1     1   Mm3
......................................................................ucggugggaGuuucguucgcuu....................   1     1   Mm3
......................................................................ucggugCgauuuucguucgcuu....................   1     1   Mm3
......................................................................uAggugggauuuucguucgcuu....................   1     1   Mm3
......................................................................ucggugggauAuucguucgcuu....................   1     1   Mm3
......................................................................ucggugggauuuucguucgcuG....................   5     1   Mm3
......................................................................ucggugggauuuucguuUgcuu....................   3     1   Mm3
......................................................................ucggugggauuuucguuAgcuu....................   6     1   Mm3
......................................................................ucggugggauuuucguucgcuuA...................   8     1   Mm3
......................................................................ucggugggauuuucguucgcuuU...................   220   1   Mm3
......................................................................ucggugggauuuucguucgcuuC...................   1     1   Mm3

.....................................................................aucggugggauuuucguGcgc......................   1     1   Fm3
.....................................................................aucggugggauuuucguucgc......................   6     0   Fm3
.....................................................................Uucggugggauuuucguucgcu.....................   1     1   Fm3
.....................................................................aucggugggauuuucguAcgcu.....................   2     1   Fm3
.....................................................................Nucggugggauuuucguucgcu.....................   1     1   Fm3
.....................................................................aucggugggauuuucguucgcG.....................   1     1   Fm3
.....................................................................aucggugggauuuucgCucgcu.....................   1     1   Fm3
.....................................................................aucggugggauuuucguucgcu.....................   144   0   Fm3
.....................................................................aucggugggauuuucgAucgcu.....................   1     1   Fm3
.....................................................................aucggugggauuuucguCcgcu.....................   2     1   Fm3
.....................................................................aucggugggauuuucguucgcuu....................   14    0   Fm3
.....................................................................aucggugggauuuucguucgcuuU...................   1     1   Fm3
......................................................................ucggugggauuuucguucU.......................   3     1   Fm3
......................................................................ucggugggauuuucguucA.......................   2     1   Fm3
......................................................................ucggugggauuuucguucg.......................   3     0   Fm3
......................................................................ucggugggauuuucguucgU......................   2     1   Fm3
......................................................................ucggugggauuuucguucgc......................   13    0   Fm3
......................................................................ucggugggauuuucguucgcu.....................   135   0   Fm3
......................................................................ucggugggauuuucguucgcuu....................   849   0   Fm3
......................................................................ucggugggUuuuucguucgcuu....................   1     1   Fm3
......................................................................ucggugggauuuucguGcgcuu....................   1     1   Fm3
......................................................................ucggugggauAuucguucgcuu....................   3     1   Fm3
......................................................................ucggugggauuuucguuGgcuu....................   4     1   Fm3
......................................................................ucggugggauuuucguuUgcuu....................   4     1   Fm3
......................................................................ucggugggauuuucguucUcuu....................   2     1   Fm3
```

Star Mature

```
cucaaacaaggauggugagcgcuacuuguuucguccggaugaaagguucggccagucuugauauucacgaucggugggauuuucguucgcuuggaauaaguagcaguccuuc
......................................................................ucggGgggauuuucguucgcuu....................   1   1   Fm3
......................................................................ucggugggauuuucguCcgcuu....................   3   1   Fm3
......................................................................ucggugggauuuucgCucgcuu....................   1   1   Fm3
......................................................................ucggugggauuuucguucgcuA....................   4   1   Fm3
......................................................................ucggCgggauuuucguucgcuu....................   1   1   Fm3
......................................................................ucggugggauuuucguucgcCu....................   1   1   Fm3
......................................................................ucggugggauGuucguucgcuu....................   1   1   Fm3
......................................................................ucggugggauuuucguAcgcuu....................   3   1   Fm3
......................................................................Ccggugggauuuucguucgcuu....................   1   1   Fm3
......................................................................ucggugggauuuucguuAgcuu....................   5   1   Fm3
......................................................................ucggugggauuuucguucgcuuU...................   23  1   Fm3
......................................................................ucggugggauuuucguucgcuuA...................   1   1   Fm3
.......................................................................cggugggauuuucguucgcuu....................   3   0   Fm3
```
